# Acute and Chronic Toxicity of Soluble Fractions of Industrial Solid Wastes on *Daphnia magna* and *Vibrio fischeri*

**DOI:** 10.1100/2012/643904

**Published:** 2012-04-22

**Authors:** Letícia Flohr, Armando Borges de Castilhos Júnior, William Gerson Matias

**Affiliations:** ^1^Laboratório de Toxicologia Ambiental, Departamento de Engenharia Sanitária e Ambiental, Universidade Federal de Santa Catarina, Campus Universitário, Caixa Postal 476, Florianópolis 88040-970, SC, Brazil; ^2^Laboratório de Pesquisas em Resíduos Sólidos, Departamento de Engenharia Sanitária e Ambiental, Universidade Federal de Santa Catarina, Florianópolis 88040-970, SC, Brazil

## Abstract

Industrial wastes may produce leachates that can contaminate the aquatic ecosystem. Toxicity testing in acute and chronic levels is essential to assess environmental risks from the soluble fractions of these wastes, since only chemical analysis may not be adequate to classify the hazard of an industrial waste. In this study, ten samples of solid wastes from textile, metal-mechanic, and pulp and paper industries were analyzed by acute and chronic toxicity tests with *Daphnia magna* and *Vibrio fischeri*. A metal-mechanic waste (sample MM3) induced the highest toxicity level to *Daphnia magna*(CE_50,48 h_ = 2.21%). A textile waste induced the highest toxicity level to *Vibrio fischeri* (sample TX2, CE_50,30 min_ = 12.08%). All samples of pulp and paper wastes, and a textile waste (sample TX2) induced chronic effects on reproduction, length, and longevity of *Daphnia magna*. These results could serve as an alert about the environmental risks of an inadequate waste classification method.

## 1. Introduction

Industrial processes generate wastes that must be adequately treated before final disposal, since the environmental risk involved is very important. An inappropriate industrial waste treatment and disposal releases toxic compounds that can contaminate the ground and water bodies [1].

 Standardized methods, like the Brazilian Waste Classification Method-NBR 10004 [2], characterizes the hazard of industrial wastes based only on chemical analysis, or on toxicity tests with mammals. When considering environmental safety in aquatic ecosystems, these methods may be inadequate, because industrial solid wastes are complex mixtures composed of various toxic substances [3, 4]. Even if each one of these compounds is present in low concentrations, synergistic and additive effects can potentiate the toxicity of mixtures [5, 6]. Therefore, the most appropriate way to evaluate toxic effects of the bioavailable fraction of wastes would be by bioassays [7].

 Aquatic environmental bioindicators like bacteria and microcrustaceans can demonstrate the toxicity potential of industrial effluents and waste leachates without the necessity of knowing all chemical substances presented in the sample [8, 9]. Toxicity effects are observed on biological parameters such as mortality, growth, and reproduction [10–12]. The observed effects are statistically analyzed and the results are expressed in numerical units, such as EC_50_ and NOEC [13].

 Taking into account the great production of solid waste in industrial activities and the environmental risk involved in its generation, treatment, and disposal, this study aims to assess the acute and chronic toxicity of soluble fraction of industrial waste samples using the test organisms *Daphnia magna* and *Vibrio fischeri*. These results could help to improve the current Brazilian Waste Classification Method, by proposing a battery of bioassays that may provide a more complete evaluation of industrial wastes hazard.

## 2. Materials and Methods

### 2.1. Waste Sampling

Field sampling was performed according to the procedures of the NBR 10007 [14], and laboratory sampling was based on the Leaching Flowchart—Appendix B—NBR 10005 [15]. Ten samples, each one from a different industry, were collected at the entrance of an industrial and sanitary landfill located in the city of Blumenau, SC, Brazil. Before entering the landfill, each sample must be previously classified according to NBR 10004 method [1]. APHA methodologies [16] are used to obtain the chemical characterization of these wastes. Different samples of the same type of waste were chosen according to their chemical characterization. Only samples of the same type of waste with very close chemical composition were collected, so it was possible to obtain the average values of chemical parameters from each type of waste. Sample characteristics are described in Table 1, according to their waste classification report.

### 2.2. Samples Preparation

Samples preparation was carried based on the NBR 10005 [14], with modifications. During this process, pH was not corrected in order to maintain the original chemical characteristics of the samples analyzed. 100 g of raw sample were introduced in a flask and filled with 2.0 L of distilled water, and the bottle was stirred for 18 hours with a rotation of 30 rpm in a rotary shaker. After stirring, the samples had their pH and dissolved oxygen (DO) measured, and the supernatant was used to carry out the toxicity tests.

### 2.3. Toxicity Tests

#### 2.3.1. *Daphnia magna* Cultivation

 Cultivation of *Daphnia magna *Straus, 1820, was performed according to ISO 6341 [17] and DIN 38412-30 [18]. Beakers with a capacity of 1500 mL of M4 culture medium were used for the growth of 25 to 30 individuals. The organisms were fed with *Scenedesmus subspicatus* algal culture, produced under ISO 8692 [19], in the CHU culture medium [20]. Cultures were kept under temperature controlled at 20 ± 2°C, 16-hour light photoperiod, and DO ≥ 2.0 mg/L.

#### 2.3.2. *Daphnia magna* Acute Toxicity Test

 Acute toxicity tests were performed according to NBR 12713 [21]. Samples were diluted with ISO medium, according to ISO 6341 [17] in four concentrations: 100, 50, 25, and 12.5%. For each concentration, 50 mL of diluted sample was prepared, and then, the solution was separated in two beakers, each one with 25 mL of the dilution. In each beaker, 10 young *D. magna* (between 2 and 26 hours of life) were inserted, resulting in an observation of the effects on immobility of 20 individuals. Organisms were exposed to these conditions for 48 hours without food and light, controlled temperature of 20 ± 2°C and OD ≥ 2.0 mg/L. 20 organisms of control group were exposed to ISO medium and kept under the same environmental conditions of the cultivated organisms. According to EPA 821-R-02-012 [22], the Trimmed Spearman-Karber Method was used to calculate CE_50,48 h_.

#### 2.3.3. *Daphnia magna* Chronic Toxicity Test

 21 days chronic toxicity tests were performed according to ISO 10706 [23], Bianchinni and Wood [24], and Knops at al. [9], with modifications. The following endpoints were analyzed: fecundity (neonates number), longevity (surviving organisms), and growth (organisms length). Each test was conducted with four sample dilutions, and a negative control (M4 medium). The dilutions were prepared according to the results observed in acute toxicity tests, and in a short chronic toxicity test of 6 days. Sample's dilution followed a geometric progression model in the ratio of 2, except for samples MM1 and MM3, and was prepared at the time of organism exposure, using appropriate proportions of sample and reconstituted water (M4 medium). For each dilution, 10 replicates were used, disposing 10 young *Daphnia magna *in 50 mL individual beakers. Each beaker received an aliquot of 25 mL test solution and was covered with plastic wrap to prevent evaporation and contamination with any residue suspended in the air. The tests were kept under the same environmental conditions of the cultivated organisms (16-hour light photoperiod, temperature 20 ± 2°C, and OD ≥ 2.0 mg/L). *D. magna* were fed with *Scenedesmus subspicatus* algae, and the media were changed every 48 hours. The organisms were checked daily and the number of survival organisms and living neonates were registered. According to EPA 821-R-02-013 [25], the results were analyzed using Dunett tests or *t*-test with Bonferroni adjustment, through the Dunnett Program version 1.5 [26]. A significance level of *P* < 0.05 was accepted.

#### 2.3.4. *Vibrio fischeri* Toxicity Test

Toxicity tests with marine luminescent bacteria *Vibrio fischeri* were performed according to ISO 11348-3 [27], and according to the methodology developed for the equipment Microtox 500 [28]. The principle used for the determination of toxicity is the inhibition of luminescence emitted by the bacteria when in contact with the sample. Exposure time was 30 minutes, and EC_50,30 min⁡_ (%) was calculated by the equipment's software.

## 3. Results

### 3.1. Physicochemical Characteristics of Industrial Wastes

 According to the composition of wastes presented in Table 1, barium, cadmium, chromium, iron, lead, and zinc are the principal components of the soluble fractions of textile wastes. The chemical analysis of the dry weight portion indicates that despite metals, cyanides and phenol also compose the textile wastes. Chemical characterization of metal-mechanical wastes indicates that chlorides, chromium, iron, and zinc are components of the dry weight portion. Zinc is the main component of the soluble fraction of these wastes. The dry weight portions of samples of pulp and paper wastes are composed by arsenite, lead, chromium, phenol, and mercury. Cadmium, lead, and chromium are the main components of the soluble fraction.

### 3.2. *Daphnia magna* and *Vibrio fischeri* Acute Toxicity Tests

 The results of *Daphnia magna *and *Vibrio fischeri* acute toxicity tests are presented in Table 2.

All samples of soluble fractions of industrial solid waste had pH and dissolved oxygen (DO) value in accordance with the recommendations for acute toxicity tests with *Daphnia magna *(pH between 5.0 and 9.0, DO ≥ 2.0 mg/L). All bacteria batches used in toxicity tests were sensitive to zinc sulfate (ZnSO_4_·7H_2_O) and correction factor (fk) within optimal values for the feasibility of the toxicity tests. Samples of soluble fraction of textile waste induced varied acute toxicity effects to both *Daphnia magna *and *Vibrio fischeri*. Samples TX3 and TX4 induced toxicity effects to both organisms tested, while sample TX2 induced no toxicity to *Daphnia magna*, and sample TX1 induced no toxicity to *Vibrio fischeri*. Sample TX1 induced little acute toxicity effects to *D. magna*, since EC_50,48 h_ = 70.71%. Sample TX2 induced toxicity effects only to *V. fischeri*, since EC_50,30 min⁡_ = 43.77%. Sample MM3, soluble fraction of metal-mechanic waste, induced the highest toxicity value of all ten samples. At the end of 48 hours of testing, all organisms observed were immobile. Sample MM2 induced toxicity effects to *V. fischeri* but did not induced serious toxicity effects to *D. magna*. Sample MM1 did not induce significative toxicity effects in these same organisms. Soluble fractions of metal-mechanic waste induced to values of EC_50,30 min⁡_ quite varied, no sample showed similar toxicity. Samples PP1 and PP2, soluble fraction of pulp and paper mill waste, induced no toxicity to *Daphnia magna*, since EC_50,48 h_ > 100%. Sample PP1 induced little toxicity to *Vibrio fischeri* (EC_50,30 min⁡_ = 91.93%). Sample PP3 induced acute toxicity effects to both *D. magna* and *V. fischeri*.

### 3.3. *Daphnia magna* Chronic Toxicity Tests

#### 3.3.1. Soluble Fraction of Textile Waste

 In Table 3, it is possible to note that only one sample of soluble fraction of textile waste induced no chronic effect. The remaining samples induced toxic effects in at least one of the observed concentrations.

Samples TX1 and TX4 induced no effects on *Daphnia magna *reproduction, since neonates mean was not significantly different than those of control for any concentrations tested. In organisms exposed to sample TX2, the neonates mean was significantly higher than the control at 12.5 and 25% concentrations. Sample TX3 induced significant chronic effects to reproduction in all concentrations tested, increasing the number of neonates. Longevity effects were induced by samples TX2 and TX4. In sample TX2, at 12.5% concentration, only one organism survived after 21 days, and at 25% concentration, no organism survived until the end of the test. In organisms exposed to sample TX4, effects were observed at 12.5% concentration, where only four organisms survived until the end of the test. Mortality was reported for organisms exposed to samples TX1 and TX3, but these do not represent a significantly lower mean than that observed in the control. Organisms' growth was also evaluated after 21 days of testing and only sample TX2 (at 6.25% concentration) induced significant growth effects on *Daphnia magna*. These organisms had a growth mean higher than those of the control group.

#### 3.3.2. Soluble Fraction of Metal-Mechanic Waste

Table 4 indicates the results regarding the effects on *Daphnia magna *exposed to soluble fractions of metal-mechanic waste in samples MM1, MM2, and MM3.

In organisms exposed to sample MM1, the neonates mean was significantly higher when compared to control (at 1.56 and 1.04% concentrations). Sample MM3 induced reproduction effects for all concentrations tested. Sample MM2 induced significant increases in reproduction at 25% concentration, and significant decreases at 50% concentration. Even without generating significant number of neonates, all organisms at 50% concentration showed eggs at the end of the test, indicating a possible delay in the development of reproductive organs of *D. magna*. The number of neonate increases at 6.25 and 12.5% concentrations, but this was not significantly higher than those of the control. Longevity of *Daphnia magna *exposed to all samples of soluble fractions of metal-mechanic waste was not significantly affected. Most organisms survived the 21-day trial. Sample MM1 induced no growth effects on *Daphnia magna*. In organisms exposed to sample MM3 (at 1.56% concentration), there was a significant length increase. Organisms exposed at 50% concentration of sample MM2 (the highest concentration tested) had a significantly lower growth in relation to the control group.

#### 3.3.3. Soluble Fraction of Pulp and Paper Waste

Table 5 indicates the results regarding to the effects on *D. magna* exposed to soluble fractions of pulp and paper mill waste in samples PP1, PP2, and PP3.

 Sample PP1 induced *Daphnia magna *reproduction effects. Significant increases in the number of neonates were observed at all concentrations, except at 100%. In organisms exposed to sample PP2, it was noted that the number of neonates produced at 12.5, 25, and 50% concentrations were higher than the control group. At 100% concentration, number of neonates was significantly lower than those of control. All samples induced longevity effects on *Daphnia magna *exposed to the highest concentration tested. In sample PP1, only three organisms at 100% concentration survived after 21 days. In sample PP2, only 4 organisms of 100% concentration survived after 21 days, and in sample PP3, no organisms of the highest dilution (50%) survived at the end of the test. All samples induced growth effects on *Daphnia magna*. In organisms exposed to samples PP1 and PP2, the effects were observed at 100% concentration. Sample PP3 induced growth effects in all concentrations tested.

## 4. Discussion

Chemical analysis confirms the presence of toxic components in the soluble fractions of all wastes analyzed, but the results of bioassays show that they induced varied toxicity effects to both *Daphnia magna *and *Vibrio fischeri*. In textile wastes, results observed in the organisms exposed to samples TX1 and TX2 can be compared to those reported by Sponza and Isik [29] and Rosa et al. [30]. These researchers observed that textile effluent and textile sludge did not induce acute toxicity effects in *D. magna* and *V. fischeri*, respectively. They concluded that the nontoxic effects were related to the efficiency of the textile treatment plant and textile sludge stabilization.

These results could also be related to antagonistic effects of cadmium, lead, and zinc [31]. However, Soetaert et al. [32] observed effects at the molecular level in *Daphnia magna *exposed to sublethal concentrations of cadmium for 48 hours: digestion, oxygen transport, cuticula metabolism, and embryo development were affected. Data related with immune and stress response, cell adhesion, visual perception, and signal transduction were also found by these researchers. Samples TX3 and TX4 induced acute toxicity effects on both organisms tested and these results can be related to the presence of metals like lead and zinc [33]. The result of acute toxicity to *D. magna *found for sample TX3 can be compared to those observed by Villegas-Navarro et al. [34], who evaluated the acute toxicity to *D. magna *of five textile effluents and found values of CE_50,48 h_ between 66.66 and 13.89% at the exit of the effluent treatment system. Researchers also observed physicochemical parameters such as hardness, conductivity, and pH and concluded that these factors may have contributed to the effluents toxicity. Nevertheless, they have not discarded the influence of other factors that may induce toxicity. Sample TX4 induced an acute toxicity to *Daphnia magna *that can be compared with the results observed by Rosa et al. [30], who analyzed the acute toxicity of fresh sludge from a textile treatment plant and observed CE_50,48 h_ = 47.88%. Researchers also found high concentrations of phenol, aluminum, and iron in these samples. Although the concentrations of aluminum and phenol in the soluble fraction of our samples were not analyzed, one can relate the acute toxicity effects with the possible presence of these substances. Sample TX4 induced toxicity effects in *V. fischeri *that can be compared to those reported by Grinevicius et al. [35]. The researchers analyzed the untreated effluent from a textile industry and found that EC_50,15 min⁡_ was 10.64% for *Vibrio fischeri*. They concluded that the untreated effluent toxicity can be linked with the high amounts of dyes, metals, surfactants, fixing, and bleaching agents that are used in the various proceedings in the textile industry.

 In the chronic toxicity investigations with *D. magna*, it is interesting to note that despite the evidence of acute toxicity induced by sample TX4, there were no toxicity effects on *D. magna'*s reproduction in the dilutions tested. Sample TX1 also did not induce chronic toxicity effects to *D. magna*. Knops et al. [9] exposed *Daphnia magna *to cetyltrimethylammonium bromide (CTAB), a cationic surfactant used in textile processing as an effluent color remover [36], and reproduction was not significantly affected. Even observing oxygen consumption decreases, and body size reductions, researchers were unable to relate these factors with effects on reproduction. In organisms exposed to sample TX2, the concentrations that showed significant increases in reproduction were the same that showed high mortality of organisms at the end of the test. Reproduction increases followed by high mortality would explain a forced attempt of species conservation [37]. The high mortality observed after 21 days exposition to sample TX2 (at 12.5 and 25% dilutions) may have been caused by the presence of metals like lead and zinc (Table 1). According to Cooper et al. [33], Zn (0.0179 mg/L) caused 90% of mortality, and Pb (0.2164 mg/L) caused 70% of mortality on *C. dubia* after a 7 days exposition. The combination of these two substances caused 60% of mortality on *C. dubia* after the same period of exposition [33]. Textile sludge contains nitrogen, phosphorous, and potassium, and these nutrients may induce growth increases in plants, algae, and earthworms [30]. This same stimulating potential may explain growth effects observed when *D. magna* was exposed to sample TX2.

 A metal-mechanic waste (sample MM3) induced the highest acute toxicity value of all ten samples analyzed. Lambolez et al. [38] also found high toxicity effects in *Daphnia magna *and *Photobacterium phosphoreum* induced by a metal slag leachate. It was considered the most toxic of all samples analyzed, and also the induced mutagenic effects in *R. subcapitata*. Kang et al. [39] evaluated the toxicity induced by effluents from an electronics factory in *Daphnia magna*. The final effluent induced a significant toxicity to *D. magna*, since EC_50,48 h_ was 15.38%. The researchers also conducted chemical analysis, and toxicity has been linked to salinity of sodium hypochlorite (NaOCl) from the effluent treatment process. Chlorides were also found in the dry weight portion of our samples, and the presence of this substance could explain the toxicity effects observed in organisms exposed to sample MM3. Samples MM1 and MM2 induced varied toxicity effects to both *D. magna* and *V. fischeri. *The interaction of chemical substances like lead, nickel, and zinc (Table 1) could have caused the synergistic and antagonistic effects to both organisms. Zinc affects negatively the uptake of nickel by *D. magna*; on the contrary, the presence of zinc stimulates the uptake of lead in this organism [31]. A binary mixture of lead and zinc induces synergistic effects to *V. fischeri *[40]. Picado et al. [41] found quite varied toxicity effects in *Vibrio fischeri* exposed to effluents from a metal industry. They observed metal concentration in these samples and found exceeded limit values of chromium, cooper, and iron.

 Chronic toxicity tests show that sample MM2 induced increases in reproduction at 6.25, 12.5, and 25% concentration, and significant decreases at 50% concentration. This same effect, known by hormesis, was observed by Rodriguez et al. [11], in *Daphnia magna *exposed to two metal surface coating effluents. These effluents contained metals like Ni, Zn, and Cr, and the researchers affirm that their presence may explain the reproduction effects. Our samples from soluble fraction of metal-mechanic wastes are also composed by these same metals. Even if the concentrations of these substances are not the same as those observed by Rodriguez et al. [11], it is possible to relate their presence with the observed effects. However, the processes that lead to higher quantities of neonates when they come in contact with low concentrations of pollutants must be further analyzed [11].

 Soluble fractions of pulp and paper wastes induced varied toxicity effects. The integration of toxic compounds present in sample PP1 may have produced antagonistic effects to both *D. magna *and *V. fischeri*, since these organisms were not affected. The presence of selenium (Table 1) may explain this observation. Hamilton et al. [42] related a delay in mortality of *Xyrauchen texanus* larvae, possibly due to the interaction of Se with other substances in water and food treatment. Sample PP2 induced the same effect to *D. magna*. On the contrary, sample PP3 induced acute toxicity effects to *D. magna* similar to those reported by Picado et al. [41]. These researchers reported CE_50,48 h_ = 51% for *D. magna*, and the evaluation of some physicochemical parameters showed that BOD, COD, TSS, oil, and greases exceeded the limit values of a Portuguese law. The toxicity effects induced by sample PP3 can also be related to the presence of metals in the soluble fractions of pulp and paper wastes. Even if the amount of each toxic substance is below the limits imposed by the Brazilian law NBR 10004 [2], synergistic and additive effects could be responsible for the observed toxicity [33]. Integration of toxic compounds from samples PP2 and PP3 could also have induced the toxic effects in *V. fischeri. *Our results can be compared to those observed by Kostamo and Kukkonen [43], who reported EC_50,30 min⁡_ values between 13 and 19% for *V. fischeri* exposed to samples of untreated pulp and paper mill effluent. Researchers also found high concentrations of resin acids and sterols in the total effluent sample. These components were not investigated in our work, but the dry weight portion of our samples contains phenol, which could have been solubilized and contributed to the toxicity effects.

 Reproduction effects were observed in organisms exposed to sample PP2. It was noted that the number of neonates produced at 12.5, 25, and 50% concentrations were higher than the control group. At 100% concentration, number of neonates was significantly lower than those of control. These results indicate hormesis in sample PP2, similar to that found by Middaugh et al. [44], who detected the same effect in the reproduction of *Ceriodaphnia dubia* during 7 days of exposure to a pulp and paper mill effluent. Researchers affirm that hormesis can be related to nutritional components presented in these effluents. Biologically treated bleached kraft pulp mill effluent contains phosphorous and carbon [45]. These nutrients could have influenced *D. magna*'s reproduction effects induced by samples PP1 and PP2. Sarakinos and Rasmussen [46] observed these same mortality effects in *Ceriodaphnia dubia* exposed to water from a river that receives pulp and paper mill effluents. After a 7 days chronic survival test, reported value of EC_50_ was 23.9%, and the researchers affirm that effects may be attributed to BOD, suspend solids, and different exposure routes, like nutrition. The presence of lead and chromium in the soluble fractions of samples PP1 and PP2 may also have contributed to the mortality observed. According to Seco et al. [47], the sublethal concentrations of these two metals for *D. magna *are 0.55 mg/L for Pb and 0.043 mg/L for Cr. Table 1 shows that in the soluble fractions of our samples these concentrations exceed the ideal limits for sublethal concentrations. All samples of pulp and paper wastes induced growth effects on *Daphnia magna*. The length of the adult organism decreased as concentration increased. These results can be compared to those reported by Knops et al. [9], who exposed *D. magna* to different cadmium concentrations, and significant length decreases were observed at the higher dilutions. Chemical characterization of our samples from pulp and paper wastes indicates the presence of cadmium, therefore, growth effects induced by sample MM2 could be attributed to this substance.

 Our results indicate that there is a considerable variability between the different waste toxicity, and also between samples of the same type of waste. Lambolez et al. [38] made these same observations and affirmed that the waste's type cannot inform about toxicity effects from the leachates.

 When comparing our toxicity tests results with the Brazilian Waste Classification Method—NBR 10004 [2], it is possible to note that, despite the fact that all wastes were classified as Class II A (nonhazardous and noninert), only sample TX1 induced low acute toxicity and no chronic toxicity effects to the organisms tested. According to NBR 10004, only Class I wastes may exhibit the toxicity characteristic. This leads to the thought that maybe this classification method has an underestimated toxicity assessment, disregarding the harmful effects of industrial landfill toxic loads in aquatic ecosystems. Similar results were reported by Silva et al. [48], who applied a battery of acute toxicity tests with *Scenedesmus subspicatus, Vibrio fischeri*, and *Daphnia magna* to textile, metal-mechanics, and automotive solid wastes. The researchers observed that in some cases the hazardous potential of industrial sludge was underestimated, so they affirm that the current Brazilian regulation (NBR 10004) is not always appropriate to evaluate the environmental impact from a solid waste. Thus, we can conclude that the use of chemical characterization to promote wastes classification is outdated and must be reviewed. Synergistic, additive, and antagonistic effects of complex mixtures cannot be predicted only by chemical analyses, and bioassays are more appropriate to effectively demonstrate toxicity levels in aquatic environments. Therefore, they should be included in the Brazilian methodologies for waste classification.

 When comparing acute and chronic toxicity tests, it is possible to confirm the great importance of chronic tests with *Daphnia magna, *since six of ten samples showed no significant acute effects but presented chronic effects in at least one of the observed variables (reproduction, growth, and longevity). Results from the *Vibrio fischeri* toxicity tests, once again, confirm the importance of chronic tests with *Daphnia magna*. Samples MM1 and PP1 induced few toxicity effects on *V. fischeri*, but chronic effects where observed in the microcrustacean.  Table 6 summarizes results for acute and chronic effects for each sample.

 Only sample TX1, considered to have low toxicity according to the acute tests with *Vibrio fischeri* and *Daphnia magna*, induced no chronic effects. Samples TX2 and PP2 were toxic to *Vibrio fischeri*, but no acute toxicity was observed to *Daphnia magna*. However, these two samples induced chronic effects in *D. magna'*s reproduction, length, and survival. De Coen and Janssen [49] affirm that digestive enzyme activity monitored after 48 h in *Daphnia magna* may be a good indicator of chronic toxicity levels, since digestive enzyme activity increases may reveal the way the organism deals with reduced absorption of food caused by a change in the efficiency of food assimilation. This hypothesis may explain the results observed in samples TX2 and PP2. Increases in digestive enzyme activity in these organisms may have caused the reproductive and physiological changes observed. Despite this, it is not possible to discard other factors that could have induced chronic effects in these samples. In chronic toxicity tests, biological characteristics may be related to many toxicity processes: (i) effects on digestive enzyme activity can be related with the inhibition on energy consumption and acquisition, which may affect the amount of food consumed and consequently interfere on the length of adult organisms; (ii) effects in vitellogenesis may alter the number of neonates produced [50]. Endocrine-active substances are involved in various industrial processes, and especially in pulp and paper industry effluents, compounds like steroids, resinic, acids and triglycerides are responsible for chronic hormonal and physiological effects [51]. So it seems that chronic toxicity effects can be caused by multiple factors.

 Samples MM1 and MM2 induced no significant acute effects for either *V. fischeri* and *D. magna*, but some chronic effects were observed. Sample MM1 induced chronic effects on reproduction, and sample MM2, on *Daphnia magna'*s reproduction and growth. The highlight occurs for sample PP1, which did not induce acute toxicity in neither of the two organisms tested, but chronic effects were observed on *D. magna'*s reproduction, length, and survival. These same observations were made by Mendonça et al. [52], and they affirm that occurrence of chronic effects on *Daphnia magna* exposed to low concentrations is important, since effluent samples did not show great acute toxicity for any of the other organisms tested (*Vibrio fischeri*, *Pseudokircheneriella subcapitata, Thamnocephalus platyurus, Daphnia magna*, and* Lemna minor*). Without chronic toxicity testing samples MM1, MM2, and PP1 probably would not be considered toxic, and the environmental risk of these wastes would not be classified as dangerous.

 Samples TX3, TX4, MM3, and PP3 had demonstrated significant toxicity potential as observed in acute tests with *Vibrio fischeri* and *Daphnia magna*. This toxicity potential was confirmed again by chronic toxicity testing with *D. magna*. Sample MM3 induced chronic effects on reproduction and growth. Sample TX3 induced reproductive effects and sample TX4 induced longevity effects. Sample PP3 induced effects on all parameters observed.

 Results found after the application of the three types of toxicity tests showed that *Vibrio fischeri* are more sensitive than *Daphnia magna *in acute toxicity tests. Interestingly, *Vibrio fischeri* tests could predict chronic toxicity in *Daphnia magna*, in seven of ten tests, it was possible to note that when *V. fischeri* showed acute toxicity, some chronic effect was also observed. The same was observed in only four acute tests with *Daphnia magna*. So, it is important to realize the three tests together, for a more complete evaluation of toxicological effects of soluble fractions of industrial waste.

 In conclusion, considering that decision-making to improve an environmental situation requires reliable measures, the use of methods that can together assess acute and chronic effects provide a more complete evaluation of toxicological effects of soluble fractions of industrial solid wastes and warn about the environmental problems caused by an inadequate waste classification, treatment, and disposal.

## Figures and Tables

**Table 1:** Characteristics of industrial solid waste samples collected at the entrance of an industrial an sanitary landfill, as classified according to NBR 10004 (ABNT, 2004c).

Parameters analyzed (mean values)	Industrial waste description^a^					
	Sludge from textile		Sludge from metal-		Sludge from pulp and paper	
	treatment plant		mechanic treatment plant		treatment plant	
	Class II A		Class II A		Class II A	
	Samples TX1, TX2, TX3, and TX4		Samples MM1, MM2, and MM3		Samples PP1, PP2, and PP3	
	Dry weight (mg/kg)	Soluble fraction (mg/L)	Dry weight (mg/kg)	Soluble fraction (mg/L)	Dry weight (mg/kg)	Soluble fraction (mg/L)
Al	—	—	ND	—	—	—
As	38.77	ND	ND	ND	49.53	0.004
Ba	—	12.72	ND	ND	—	0.72
Cd	4.73	0.05	ND	ND	—	0.04
Pb	155.77	0.72	4.6	0.24	709.87	0.59
Cyanides	47.07	—	ND	—	—	—
Chlorides	—	—	939.5	—	—	—
Cu	—	—	ND	ND	—	—
Cr_total_	50.59	1.46	228.58	0.25	98.98	0.07
Phenol	4.61	—	8.73	—	1.2	—
Fe	—	0.05	39631.8	—	—	—
Mg	—	—	240.34	—	—	—
Mn	—	—	148.3	—	—	—
Hg	ND	ND	ND	ND	0.09	0.0001
Ni	—	—	0.63	0.13	—	—
Oil and greases	—	—	0.08	—	—	—
Ag	—	—	ND	ND	—	0.03
Se	9.2	0.15	ND	ND	4.67	0.05
Zn	—	0.1	639.22	1.45	—	—

^a^ NBR 10004 Classification Report submitted to the industrial and sanitary landfill, by industries responsible for waste.

— Not analyzed.

ND Not detected.

**Table 2:** Results of *Daphnia magna* and *Vibrio fischeri* acute toxicity tests after exposition to soluble fraction of textile, metal-mechanic, and pulp and paper industrial solid waste.

Industry	Sample	*Daphnia magna *CE_50, 48 h_ (%)	*Vibrio fischeri *CE_50, 30 min⁡_ (%)	pH	OD (mg/L)
Textile	TX1	70.71	NT	6.83	2.9
	TX2	NT	43.77	7.75	5.8
	TX3	11.26	12.08	7.71	4.0
	TX4	48.29	17.99	6.9	3.2

Metal-mechanic	MM1	84.86	73.86	8.89	4.3
	MM2	70.71	48.73	9.47	4.7
	MM3	2.21	17.47	6.9	5.8

Pulp and paper	PP1	NT	91.93	6.72	4.1
	PP2	NT	19.00	8.17	5.6
	PP3	51.76	18.61	6.3	3.4

NT Nontoxic.

**Table 3:** Effects on reproduction, longevity and growth of *Daphnia magna* exposed to soluble fractions of textile wastes, samples TX1, TX2, TX3, and TX4, during 21 days. Results are expressed as means ± standard deviation.

Sample	Concentration (%)	Reproduction: no. neonates/broods	Longevity: no. survivors	Growth: length (mm)
TX1	Control	21.9 ± 4.17	10 ± 0.0	4.3 ± 0.48
	6.25	24.6 ± 4.33	8 ± 0.42	4.57 ± 0.42
	12.5	27.91 ± 3.05	9 ± 0.32	4.71 ± 0.29
	25	26.7 ± 5.90	9 ± 0.32	4.75 ± 0.39
	50	26.24 ± 4.30	7 ± 0.48	4.6 ± 0.19

TX2	Control	21.9 ± 4.17	10 ± 0.0	4.25 ± 0.42
	3.125	19.14 ± 3.67	9 ± 0.32	4.44 ± 0.42
	6.25	30.09 ± 5.45	10 ± 0.0	4.92 ± 0.20^a^
	12.5	33.56 ± 11.78^a^	1 ± 0.32^a^	4.44 ± 0.39
	25	38.98 ± 5.25^a^	0.0 ± 0.0^a^	4.6 ± 0.42

TX3	Control	19.11 ± 2.54	10 ± 0.0	3.9 ± 0.21
	0.78	25.32 ± 4.51^a^	10 ± 0.0	3.77 ± 0.36
	1.56	30.91 ± 3.71^a^	9 ± 0.32	4.06 ± 0.32
	3.125	31.16 ± 7.11^a^	9 ± 0.32	3.87 ± 0.35
	6.25	38.14 ± 5.18^a^	7 ± 0.48	4.0 ± 0.0

TX4	Control	20.66 ± 3.03	10 ± 0.0	4.75 ± 0.35
	1.56	23.64 ± 3.92	7 ± 0.48	4.75 ± 0.42
	3.125	22.72 ± 7.22	6 ± 0.52	4.92 ± 0.19
	6.25	23.67 ± 7.84	6 ± 0.52	4.83 ± 0.25
	12.5	16.8 ± 3.27	4 ± 0.52^a^	4.66 ± 0.41

^a^ Values which differ significantly from control (*P* < 0.05).

**Table 4:** Effects on reproduction, longevity and growth of *Daphnia magna* exposed to soluble fractions of metal-mechanic waste, samples MM1, MM2, and MM3, during 21 days. Results are expressed as means ± standard deviation.

Sample	Concentration (%)	Reproduction: no. neonates/broods	Longevity: no. survivors	Growth: length (mm)
MM1	Control	18.98 ± 2.58	10 ± 0.0	3.7 ± 0.48
	0.52	22.45 ± 3.29	9 ± 0.32	3.83 ± 0.41
	0.78	20.09 ± 4.69	8 ± 0.42	3.87 ± 0.35
	1.04	24.53 ± 3.96^a^	9 ± 0.32	4 ± 0.0
	1.56	23.77 ± 4.34^a^	10 ± 0.0	4 ± 0.0

MM2	Control	15.34 ± 3.30	10 ± 0.0	3.95 ± 0.16
	6.25	16.37 ± 3.65	10 ± 0.0	3.8 ± 0.26
	12.5	20.11 ± 5.19	10 ± 0.0	3.85 ± 0.24
	25	27.49 ± 9.31^a^	10 ± 0.0	3.9 ± 0.21
	50	2.66 ± 2.08^a^	10 ± 0.0	3.65 ± 0.34^a^

MM3	Control	16.23 ± 2.58	10 ± 0.0	3.85 ± 0.24
	0.52	20.41 ± 3.41^a^	9 ± 0.32	3.93 ± 0.19
	0.78	22.18 ± 2.98^a^	8 ± 0.42	3.94 ± 0.32
	1.04	27.56 ± 1.99^a^	10 ± 0.0	3.94 ± 0.17
	1.56	26.21 ± 2.77^a^	9 ± 0.32	4.21 ± 0.27^a^

^a^ Values which differ significantly from control (*P* < 0.05).

**Table 5:** Effects on reproduction, longevity, and growth of *Daphnia magna* exposed to soluble fractions of pulp and paper mill waste, samples PP1, PP2, and PP3, during 21 days. Results are expressed as means ± standard deviation.

Sample	Concentration (%)	Reproduction: no. neonates/broods	Longevity: no. survivors	Growth: length (mm)
PP1	Control	19.57 ± 2.70	10 ± 0.0	4.1 ± 0.21
	12.5	33.27 ± 5.15^a^	9 ± 0.32	3.94 ± 0.30
	25	28.96 ± 6.15^a^	10 ± 0.0	3.9 ± 0.32
	50	29.32 ± 6.57^a^	10 ± 0.0	4.06 ± 0.42
	100	24.34 ± 9.10	3 ± 0.48^a^	3.08 ± 0.49^a^

PP2	Control	19.49 ± 3.89	10 ± 0.0	4.8 ± 0.42
	12.5	26.43 ± 3.22^a^	10 ± 0.0	4.9 ± 0.32
	25	24.84 ± 3.03^a^	10 ± 0.0	4.66 ± 0.71
	50	24.22 ± 4.76^a^	10 ± 0.0	4.9 ± 0.32
	100	5.78 ± 4.67^a^	4 ± 0.52^a^	4.25 ± 0.96^a^

PP3	Control	9.20 ± 1.74	10 ± 0.0	4.2 ± 0.26
	6.25	5.49 ± 1.92^a^	10 ± 0.0	2.95 ± 0.28^a^
	12.5	8.13 ± 2.60	10 ± 0.0	2.8 ± 0.35^a^
	25	6.81 ± 3.50	10 ± 0.0	2.55 ± 0.44^a^
	50	1.50 ± 0.71^a^	0 ± 0.0^a^	2.75 ± 0.35^a^

(^a^) Values which differ significantly from control (*P* < 0.05).

**Table 6:** Summary of acute effects on *Vibrio fischeri* and *Daphnia magna* and chronic effects (reproduction, longevity, and growth) in *Daphnia magna* exposed to soluble fractions of textile, metal-mechanic, and pulp and paper solid waste.

Industry	Sample	*V. fischeri *CE_50, 30 min⁡_ (%)	*D. magna *CE_50, 48 h_ (%)	*D. magna* Reproduction effects	*D. magna *Longevity effects	*D. magna* Growth effects
Textile	TX1	NT	70.71	NO	NO	NO
	TX2	43.77	NT	O	O	O
	TX3	12.08	11.26	O	NO	NO
	TX4	17.99	48.29	NO	O	NO

Metal-mechanic	MM1	73.86	84.86	O	NO	NO
	MM2	48.73	70.71	O	NO	O
	MM3	17.47	2.21	O	NO	O

Pulp and Paper	PP1	91.93	NT	O	O	O
	PP2	19.00	NT	O	O	O
	PP3	18.61	51.76	O	O	O

(NT) Nontoxic.

(O) Observed.

(NO) Not observed.
